# Anxiety, depression and post-traumatic stress disorder management after critical illness: a UK multi-centre prospective cohort study

**DOI:** 10.1186/s13054-020-03354-y

**Published:** 2020-11-02

**Authors:** Robert Hatch, Duncan Young, Vicki S. Barber, John Griffiths, David A. Harrison, Peter J. Watkinson

**Affiliations:** 1 Critical Care Research Group, Nuffield Department of Clinical Neurosciences, University of Oxford, Oxford, UK; 2 Oxford Clinical Trials Research Unit, University of Oxford, Oxford, UK; 3 Adult Intensive Care Unit, Oxford University Hospitals NHS Foundation Trust, Oxford, OX3 9DU UK; 4 Intensive Care National Audit and Research Centre (ICNARC), London, UK

**Keywords:** Depression, Anxiety, Post-traumatic stress disorder, Questionnaire, Mental health, Primary care, Psychopathology, Intensive care, Outcomes, Multi-centre, Critical illness

## Abstract

**Background:**

Survivors of critical illness have significant psychopathological comorbidity. The treatments offered by primary health care professionals to affected patients are unstudied.

**Aim:**

To report the psychological interventions after GPs received notification of patients who showed severe symptoms of anxiety, depression or Post-Traumatic Stress Disorder.

**Methods:**

Design: Multi-centre prospective cohort sub-study of the ICON study. Setting: NHS primary care in the United Kingdom. Participants: Adult patients, November 2006–October 2010 who had received at least 24 h of intensive care, where the general practitioner recorded notification that the patient had reported severe symptoms or caseness using the Hospital Anxiety and Depression Scale (HADS) or the Post-Traumatic Stress Disorder Check List-Civilian (PCL-C). Interventions: We notified general practitioners (GPs) by post if a patient reported severe symptoms or caseness and sent a postal questionnaire to determine interventions after notification. Main outcome measure: Primary or secondary healthcare interventions instigated by general practitioners following notification of a patient’s caseness.

**Results:**

Of the 11,726 patients, sent questionnaire packs containing HADS and PCL-C, 4361 (37%) responded. A notification of severe symptoms was sent to their GP in 25% (1112) of cases. Of notified GPs, 65% (725) responded to our postal questionnaire. Of these 37% (266) had no record of receipt of the original notification. Of the 459 patients where GPs had record of notification (the study group for this analysis), 21% (98) had pre-existing psychopathology. Of those without a pre-existing diagnosis 45% (162) received further psychological assessment or treatment. GP screening or follow-up alone occurred in 18% (64) whilst 27% (98) were referred to mental health services or received drug therapy following notification.

**Conclusions:**

Postal questionnaire identifies a burden of psychopathology in survivors of critical illness that have otherwise gone undiagnosed following discharge from an intensive care unit (ICU). After being alerted to the presence of psychological symptoms, GPs instigate treatment in 27% and augmented surveillance in 18% of cases.

**Trial registration:**

ISRCTN69112866 (assigned 02/05/2006).

## Background

Survivors of critical illness are at high risk of suffering long-term mental and psychological impairment after treatment on an intensive care unit (ICU) [1] with around 33% suffering from symptoms associated with anxiety or depression [2, 3] and 19% suffering clinician-diagnosed post-traumatic stress disorder (PTSD) [4, 5]. When symptoms of one of the three psychological disorders are present, there is a 65% chance they will co-occur with symptoms of at least one of the other two disorders [6]. Symptoms persist for at least a year after discharge from hospital [3, 5]. Mental health is a key component of health-related quality of life (HRQoL). Survivors of critical illness experience a lower HRQoL than the general population [7]. The high psychopathological burden amongst ICU survivors is potentially treatable [4], and early treatment of these conditions can result in improved HRQoL [8].

Although there are recommended pathways to treat depression [9] and PTSD [10] in a community setting, the treatments received by patients developing these conditions as a result of critical illness treated on an ICU are unknown. The ICON Study was a large multi-centre postal survey of HRQoL and psychopathology in ICU survivors across 26 centres in the UK [11]. With patients’ consent, their general practitioners (GPs) were notified when they reported symptoms of anxiety, depression or PTSD that exceeded standard thresholds on validated scoring systems.

We prospectively studied the medical management after notifying a patient’s GP to the presence of possible psychopathology following critical illness.

## Methods

We report our study following STROBE guidance [12].

### Study design

The detailed methodology of the ICON study has already been reported [6, 13, 14]. The ICON study assessed Heath-Related Quality of Life (HRQoL), symptoms of anxiety and depression (Hospital Anxiety and Depression Scale (HADS) [15]) as well as caseness of PTSD (PTSD Checklist–Civilian version (PCL-C) [16]), following 24 h or more of treatment on an ICU [2]. In this sub-study, we report all patients over the age of 16 admitted to any of 26 UK ICUs between November 2006 and October 2010 and sent questionnaire packs containing HADS and PCL-C. This study reports the effect of GP notification on psychological interventions in the subgroup of the ICON Study who showed severe symptoms. GPs had to report receipt of the notification for patients to be included in the analysis.

We sent participants postal questionnaires at 3 and 12 months after discharge from an ICU, if they had been discharged from hospital. We tracked patient mortality via written communication with their GP and use of the NHS Summary Care Record [17]. We contacted patients who left hospital and were alive 3 months following discharge from ICU in writing. We prospectively defined a HADS anxiety or depression sub-score greater than or equal to 15 as suggesting severe anxiety or depression, respectively [13]. A PCL-C score greater than or equal to 45 defined PTSD caseness [13]. We defined mixed caseness as triggering notification for PTSD and either anxiety or depression. The descriptions of these populations have already been published [6]. Data obtained included: admission diagnoses, severity of illness at ICU admission and in-ICU treatment using data linkage to the Intensive Care National Audit and Research Centre Case Mix Programme database.

### Setting and participants

This sub-study of the ICON study [6, 11, 14], included patients who reported severe symptoms from all phases of the ICON study with either a HADS or PCL-C questionnaire (phase 1 and group B of phase 2—Additional file 1: Appendix A).

### Variables: GP notification

We contacted the GP by post when a patient’s HADS or PCL-C response exceeded the pre-defined thresholds [13, 17]. The notification letter contained an explanation of the study, details of the patient’s ICU admission, and the score that prompted this action. It did not suggest or mandate further action. We notified GPs only on the first occasion a patient met pre-defined thresholds for each instrument.

### Data sources: survey of treatments offered by GPs

We surveyed GPs actions following notification for patients alive November 2011. Postal questionnaires were sent to the current GP (as denoted by the NHS Summary Care Record). Mailings contained a cover letter describing the study, a self-addressed envelope, a copy of the patients signed consent form and a single-sided A4 questionnaire. We took no further action if the survey was not returned to the study office.

Each questionnaire sent to a GP contained the patient’s name, date of birth, NHS number, ICU admission date and the name of the ICU in which the patient was treated. A list of options then followed with a tick box adjacent to each option. Respondents were encouraged to tick all options that applied. The first questions confirmed identity of the patient, awareness of their ICU admission and receipt of the notification letter alerting them to a potential case of psychological disease. Where receipt of the notification letter could not be confirmed, GP’s did not complete the rest of the questionnaire. The remaining questions were based on the work of Kendrick et al. [18] who studied the management of depression in primary care resulting from the scoring of questionnaire-based screening. The questionnaire is reproduced in Additional file 3: Appendix Form C. We used an electronic form reader (Teleform v10, Cambridge, UK) to read responses into a database (MySQL v5.0—Oracle Corporation, Redwood Shores, CA). Study office personnel entered data that the electronic form reader could not interpret.

### Bias

Eligible patients were selected automatically by the database system based solely on the scored responses to questionnaires and confirmed valid, current consent. GPs were not incentivised to participate. The patients could remove their consent at any time.

### Study size

Phase 1 and Phase 2—group B of the ICON Study contain 17,300 patients admitted to an ICU. Only patients who survived beyond 3 months after leaving ICU, responded to the study questionnaires and met pre-determined thresholds of caseness were eligible for inclusion in this study.

### Quantitative variables

The primary outcome measure was the incidence of medical action following confirmed receipt of a notification of a potential case of psychological disease. Medical treatment was defined as a patient receiving either drug therapy or a referral to any form of mental health services. Repeat screening was defined as receiving either a face-to-face consultation or other forms of clinical screening**.** Secondary outcome measures included: response rate, the incidence of pre-existing psychopathology, prior awareness of the patient’s admission to ICU and confirmation of receipt of the study notification letter. The term other psychotropic drug is used to refer to a drug of this type that is not an antidepressant or anxiolytic.

### Statistical analysis

The response rate to the GP questionnaire was defined as receipt of the GP questionnaire by the study office as a proportion of all questionnaires sent. Analysis was performed using R Core (version 3.4.1). The Chi-squared statistic was used to test the relationship between categorical variables. The responses to the medical management questions were collapsed into three groups: GP screening or follow-up, drug therapy and referral to mental health services. Referral to mental health services included a primary care mental health worker.

## Results

### Participants

Following discharge from ICU, 11,726 of 17,300 (68%) patients admitted to an ICU were alive and sent a patient questionnaire pack containing HADS and PCL-C questionnaires at 3- or 12-month post-ICU discharge. Of these, 4361 consented and responded (37%). The responses of 1112 (25%) individual consenting patients resulted in a notification being sent to their GP following a HADS or PCL-C score meeting thresholds. The majority of GPs, 832 (75%), were first notified following responses received after patient surveys sent out at 3-month post-ICU discharge (Additional file 5: Appendix E). Out of all the GP notifications sent, 0.5% of patients died between generation of notification letter and the GP taking receipt. GP notification was automated with 99.9% occurring within 7 days of receiving a patient survey response meeting notification criterion.

### Descriptive data

**Fig. 1:**
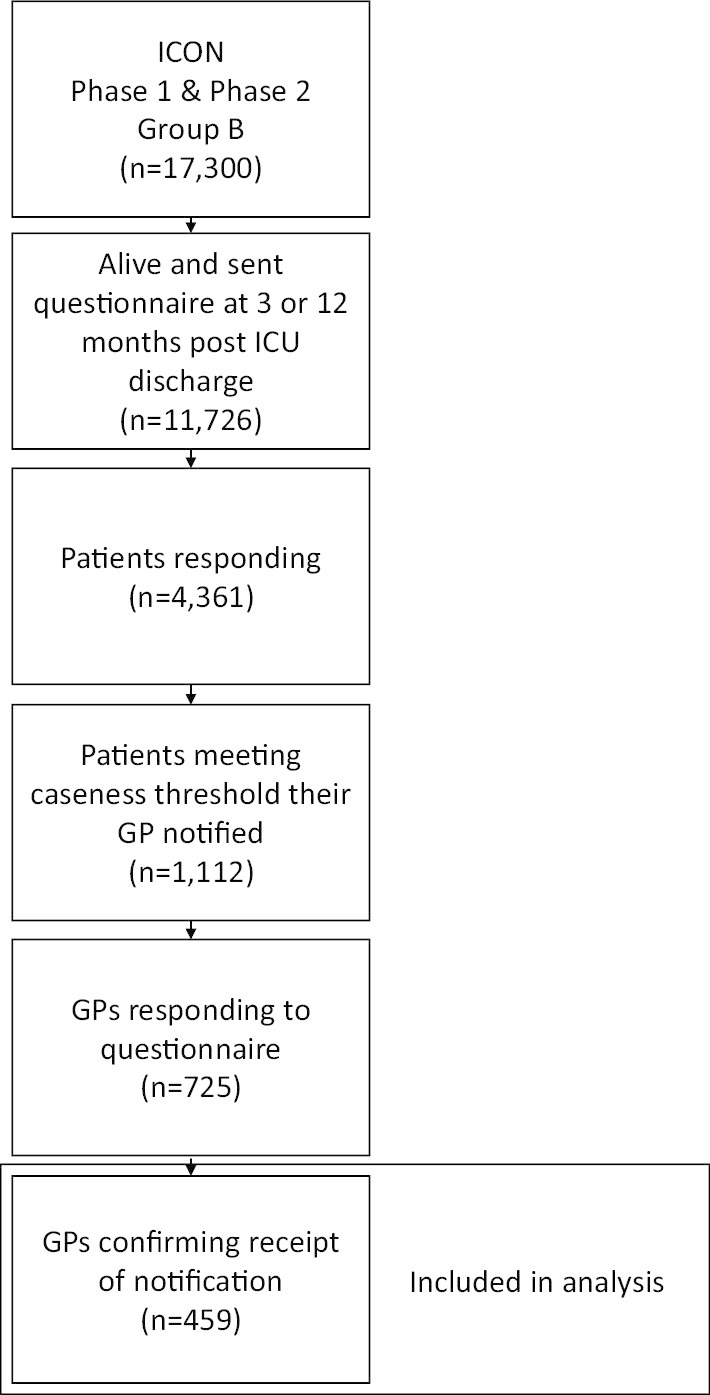
Flow diagram

Of the 725 (65%) GPs that responded to the GP questionnaire, 459 (63%) reported having received the original notification letter from the ICON Study (Fig. 1).

Table 1 shows the demographics of all patients responding to the study and those meeting the threshold of psychopathology resulting in notification of their GP.

**Table 1:** Demographics

	Responders	Suspected psychopathology/caseness
Patients *n*	4361	1112
Age, years (median) [IQR]	64 [52–74]	56 [45–67]
Sex (% male)	57%	50%
APACHE II score (median) [IQR]	15 [1–18]	14 [10–18]
ICU length of stay, days (median) [IQR]	3 [2–6]	3 [2–7]
Hospital length of stay, days (median) [IQR]	15 [9–27]	16 [8–29]
Advanced cardiac support days (median) [IQR]	0 [0–0]	0 [0–0]
Basic cardiac support days (median) [IQR]	3 [2–5]	3 [2–6]
Advanced respiratory support days (median) [IQR]	1 [0–3]	1 [0–3]
Basic respiratory support days (median) [IQR]	1 [0–2]	1 [0–3]
Renal support days (median) [IQR]	0 [0–0]	0 [0–0]
Neurological support days (median) [IQR]	0 [0–0]	0 [0–0]
Liver support days (median) [IQR]	0 [0–0]	0 [0–0]
Dermatological support days (median) [IQR]	0 [0–0]	0 [0–0]
Gastrointestinal support days (median) [IQR]	0 [0–4]	0 [0–5]
Primary reason for ICU admission (ranked by frequency, top 15)	Vascular procedure to major vessel *n* = 357 8%	Respiratory tract infection * n* = 101 9%
	Respiratory tract infection *n* = 343 8%	Self-poisoning *n* = 68 6%
	Large bowel tumour *n* = 301 7%	COPD *n* = 41 4%
	Acute renal failure *n* = 159 4%	Vascular procedure to major vessel *n* = 41 4%
	Septicaemia and septic shock *n* = 151 3%	Acute renal failure *n* = 40 4%
	Malignant neoplasm of oesophagus *n* = 137 3%	Large bowel tumour * n* = 37 3%
	COPD * n* = 134 3%	Septicaemia and septic shock * n* = 36 3%
	Self-poisoning * n* = 104 2%	Not recorded * n* = 35 3%
	Not recorded * n* = 104 2%	Asthma attack in new or known asthmatic * n* = 28 3%
	Bowel perforation * n* = 89 2%	Status * n* = 27 2%
	Acute pancreatitis * n* = 79 2%	Bowel perforation * n* = 25 2%
	Asthma attack in new or known asthmatic * n* = 74 2%	Malignant neoplasm of oesophagus * n* = 21 2%
	Status * n* = 69 2%	Acute pancreatitis * n* = 19 2%
	Ventricular tachycardia or fibrillation * n* = 57 1%	Acute myocardial infarction * n* = 12 1%
	Acute myocardial infarction * n* = 55 1%	Diabetic ketoacidosis * n* = 12 1%

### Outcome data

Amongst all patients where their GP recorded receiving the original notification (*n* = 459), the precipitating questionnaire-based alerts resulted from isolated PTSD caseness in 47% (*n* = 216), mixed psychopathology in 43% (*n* = 195) isolated depression in 5% (*n* = 25) and isolated anxiety in 5% (*n* = 23) (see Additional file 2: Appendix B).

### Main results

**Fig. 2:**
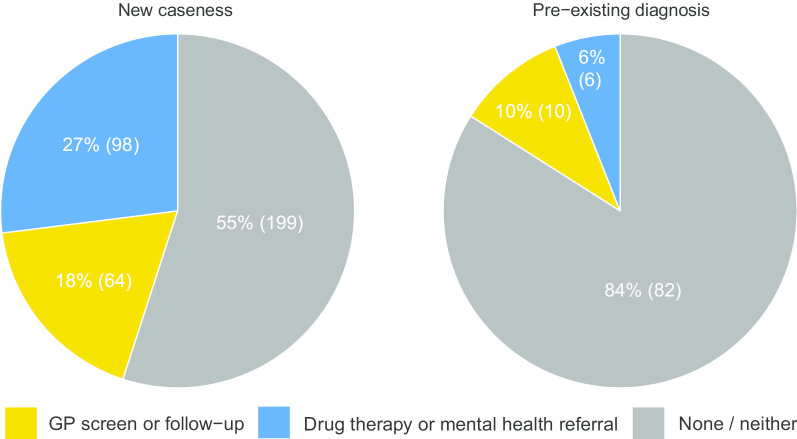
Pie chart of management outcomes

A pre-existing diagnosis of either anxiety, depression or PTSD was present in 21% (98/459) of patients at the time of receipt of the warning letter. The breakdown of patients with and without a pre-existing diagnosis compared to medical management received is shown in Fig. 2. Amongst patients displaying severe symptomatology with no prior psychological diagnosis, notification of the GP was followed by 45% (162/361) of patients receiving further assessment or treatment delivered either by the GP or in dedicated mental health service. Repeat screening or assessment by the GP with no other referral or drug therapy occurred in 18% (64/361) of individuals. Medical therapy or specialist referral occurred in the remaining 27% (98/361) of those detected (Fig. 2 and Additional file 4: Appendix D). Of these, 16% (57) were prescribed an antidepressant, 5% (19) an anxiolytic and a further 2% (8) another psychotropic drug (Table 2). Referral to counselling occurred in 8% (*n* = 28), psychiatry 3% (*n* = 12) or mental health worker 2% (*n* = 9) review (Table 2).

**Table 2:** Breakdown of management outcomes

	New caseness (*n* = 361)
GP screening	12% (*n* = 45)
GP follow-up	23% (*n* = 82)
Antidepressant drug	16% (*n* = 57)
Anxiolytic drug	5% (*n* = 19)
Other psychotropic drug	2% (*n* = 8)
Counsellor	8% (*n* = 28)
Primary care mental health worker	2% (*n* = 9)
Psychology services	2% (*n* = 7)
Social Services	1% (*n* = 2)
Psychiatry	3% (*n* = 12)

All outcomes are non-exclusive (individual patients can receive multiple management outcomes)

Table 3 further subdivides these management outcomes by caseness subgroup, amongst those patients with no prior diagnosis of anxiety, depression or PTSD.

**Table 3:** Breakdown of management outcomes by psychopathology subtype amongst those without a prior diagnosis

	Anxiety or depression 13% (*n* = 46)	PTSD 52% (*n* = 188)	Mixed 35% (*n* = 127)
GP screening	11% (*n* = 5)	12% (*n* = 23)	13% (*n* = 17)
GP follow-up	24% (*n* = 11)	20% (*n* = 37)	27% (*n* = 34)
Antidepressant drug	22% (*n* = 10)	12% (*n* = 22)	20% (*n* = 25)
Anxiolytic drug	9% (*n* = 4)	3% (*n* = 6)	7% (*n* = 9)
Other psychotropic drug	2% (*n* = 1)	1% (*n* = 1)	5% (*n* = 6)
Counsellor	7% (*n* = 3)	7% (*n* = 13)	9% (*n* = 12)
Primary care mental health worker	0% (*n* = 0)	2% (*n* = 4)	4% (*n* = 5)
Psychology services	2% (*n* = 1)	3% (*n* = 6)	0% (*n* = 0)
Social Services	2% (*n* = 1)	1% (*n* = 1)	0% (*n* = 0)
Psychiatry	7% (*n* = 3)	1% (*n* = 2)	6% (*n* = 7)

All outcomes are non-exclusive (individual patients can receive multiple management outcomes)

No further action after receipt of the notification letter occurred much more frequently in those with pre-existing disease than those without (82/98 versus 199/361, *p* < 0.001) Fig. 2.

## Discussion

### Key results

This is the first study describing the management of PTSD, anxiety and depression identified using a postal survey of survivors of critical illness. Nearly one quarter of patients who reported symptoms of anxiety, depression or PTSD following critical illness had a psychological diagnosis that pre-dated the first (3 month) questionnaire. As this was prior to the notifications being sent to GPs, the study itself could not have influenced diagnosis. However, in the three quarters without a pre-existing diagnosis a significant proportion (27%) received medical therapy or specialist referral. Without these notifications, some of these patients may have presented at the GP, but the notifications may have increased the timeliness of treatments.

### Limitations

We have used two separate postal survey questionnaires. First, to assess the symptom burden amongst participants and then subsequently, as a means of assessing the outcome resulting from written notification of their GP. Survey questionnaires are subject to many biases and using two in series is likely to incur a double selection bias [19]. Selection bias is well described amongst responders to postal questionnaires [20, 21]. Following critical illness, it is also possible that the physical and mental barriers involved in responding to the study office may exert a degree of attrition bias from the outset. Response bias in the forms of acquiescence bias and extreme responding are also well-recognised features of postal questionnaires. However, from a pragmatic perspective, our question relates to the diagnostic yield of completed postal screening amongst those that do respond, rather than those that are lost to follow-up.

The determinants of response by GPs to our questionnaire are also subject to various forms of response bias. GPs were not incentivised to respond, differences in practice management, resourcing, record keeping and research involvement are all likely factors that will have influenced response. Despite these limitations, postal survey is a relatively unobtrusive, cost effective and efficient means of collecting data [20]. The instruments used to identify the three causes of psychopathology are well validated.

We know from the ICON study and similar smaller studies that postal survey identifies the symptoms associated with psychological disease [6, 14, 21]. The relativity low response rate amongst these studies means their results may not be representative of all survivors. It may also be that sufferers are over-represented in the responders (volunteer bias) [14]. However, considering the relative ease and low cost associated with performing a postal survey, and the fact that three quarters of caseness was newly identified before the patient consulted their GP, it may allow early identification and intervention in many sufferers. In this sub-study, the proportions of patients with symptoms of PTSD versus anxiety/depression are higher than in previous ICON publications as the study design used a higher threshold applied to both HADS sub-scores to define severe symptoms than the lower caseness threshold used previously.

An overall response rate of 66% amongst GPs is as expected especially considering there was no obligation or incentive [22]. That 37% of those who responded could not identify the original warning letter is certainly of concern and suggests the style or type of notification needs review. Failure of the postal services seems unlikely at this scale and did not occur in the ICON study when sending questionnaires to participants who had already responded. There are known to be large variations in the accuracy and completeness of the clinical information stored in electronic patient records [23]. It is possible that the interface between paper notification letters and progressively more electronic general practice records contritubuted to failure to find the original letter. Regardless of the explanation for this result, this study highlights some of the challenges involved in establishing high-quality exchange of medical data between interested parties.

Both instruments used have been validated and their psychometric properties described. HADS has been used extensively in both survivors of critical illness and much wider patient populations. Alerting was based on the definition of `severe` symptoms (score ≥ 15). However, systematic reviews of the use of HADS have shown that using the much lower cut-off value of (≥ 8) is associated with a specificity and sensitivity of 0.8 [24]. This may account for the relatively high psychopathological burden and subsequent interventions amongst those with anxiety and depression. It also suggests that there is a significant unstudied population of patients with anxiety and depression within the ICON dataset.

Conversely, PCL-C has been predominantly used outside of the post ICU setting. A correlation of 0.93 between the total PCL score and structured interview with the Clinician-Administered PTSD Scale (CAPS) has been demonstrated with a diagnostic efficiency of 0.9 versus the CAPS [25]. A score of 45 or greater on the PCL-C has been recommended as a cut off point for high PTSD symptom load [13]. However, this can vary between populations, for example a threshold of 50 in breast cancer patients resulted in a sensitivity of 0.60 and a specificity of 0.99 [13]. Regardless, of the threshold chosen, any instrument will ultimately result in a dichotomous outcome at the point of deciding whether to act upon the result. If PCL-C were to be used as a screening tool further work would be required in order to evaluate its yield and potential benefit/harm as well as cost.

### Interpretation

GP intervention following notification of severe symptoms reported in a HADS or PCL-C questionnaire depends markedly on prior knowledge of disease. Receipt of an alert in a patient with a pre-existing diagnosis was followed by only 6% receiving further intervention. In contrast, in those with no prior diagnosis, nearly half received further assessment, with 27% receiving additional therapy or mental health referral. Our findings suggest there is a significant unmet psychopathological need in survivors of critical illness that routine use of HADS or PCL-C questionnaires could help detect.

### Generalisability and future work

Our study was undertaken throughout the United Kingdom, suggesting our results will be generalisable, within the limits of our postal questionnaire approach. Responders to postal questionnaire may not be representative of the entire population of survivors of critical illness due to reporting as well as other biases however, we clearly define an at-risk group that required further attention at the point of GP notification. Further research is required to establish whether identification of psychopathology by questionnaire survey could lead to improved outcomes for survivors of critical care. This work may be particularly important given the increased mortality associated with symptoms of depression following critical illness [23].

## Conclusions

Postal survey detects a burden of psychopathological disease in around a quarter of those who have survived a period of ICU treatment and return a questionnaire. In those without a prior diagnosis, GP notification of questionnaire-identified severe symptomatology was followed by GP medical treatment or mental health referral in 27% of patients.

## Supplementary information

**Additional file 1** Phases and instrument breakdown.

**Additional file 2** Venn diagram of reason for GP warning letters.

**Additional file 3** GP postal questionnaire.

**Additional file 4** Management outcomes (Figure 2 as table).

**Additional file 5** Response rates.

## Data Availability

The anonymised dataset is available from the corresponding author. We did not obtain consent for data sharing, so we will only make anonymised aggregated data available.

## Abbreviations

EQ-5D-3L: EuroQol group 5 dimensions 3 level
HADS: Hospital Anxiety and Depression Scale
ICNARC: Intensive Care National Audit and Research Centre
ICON: Intensive Care Outcome Network
ICU: Intensive Care Unit
PCL-C: Post-Traumatic Stress Disorder Check List-Civilian
REC: Research Ethics Committee
SF36v2: Short form 36 version 2
SWAT: Study within a trial
UK: United Kingdom
