# Supplementary figures and images for: Anxiety, depression and post-traumatic stress disorder management after critical illness: a UK multi-centre prospective cohort study
Source: Crit Care. 2020 Nov 2;24:633. doi: 10.1186/s13054-020-03354-y (PMC7607621; doi:10.1186/s13054-020-03354-y)

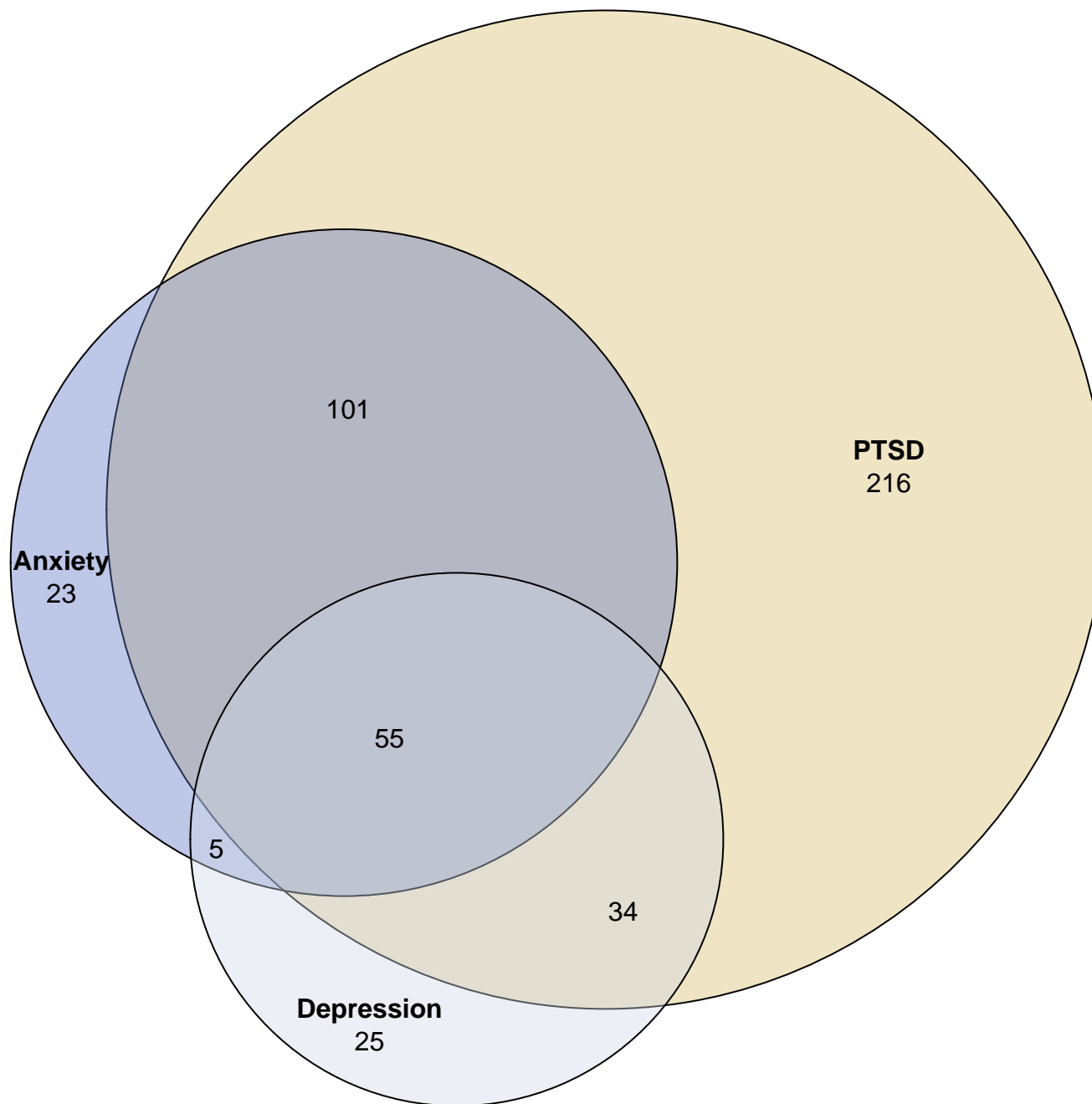

Supplement: Supplementary file 2 — Additional file 2 Venn diagram of reason for GP warning letters. [file 13054_2020_3354_MOESM2_ESM.pdf]
