# Supplementary material for: Anxiety, depression and post-traumatic stress disorder management after critical illness: a UK multi-centre prospective cohort study
Source: Crit Care. 2020 Nov 2;24:633. doi: 10.1186/s13054-020-03354-y (PMC7607621; doi:10.1186/s13054-020-03354-y)
Supplement: Supplementary file 3 — Additional file 3 GP postal questionnaire. [file 13054_2020_3354_MOESM3_ESM.pdf]

GP QUESTIONNAIRE

26141

G	A						

Intensive Care Outcome Network study

Thank you for taking the time to complete this short questionnaire. Please answer all the questions to the best of your knowledge, leaving unanswerable questions blank.

Patient Details:

Following ICU admission on the 1/1/1970

Were you aware that your patient was admitted to the ICU at the ABC Hospital on the 1/1/1970?

☐ Yes ☐ No

Did you receive a letter from the ICON study informing you that your patient was at risk of depression / anxiety / post traumatic stress disorder?

☐ Yes ☐ No

If No, thank you for completing this questionnaire

Did the letter from the ICON study warning of your patient's possible depression / anxiety / post traumatic stress disorder prompt further treatment? (please tick all those that apply)

- ☐ Repeat psychological screening assessment
- ☐ Follow-up appointment within 4 weeks time
- ☐ Prescription of an antidepressant drug
- ☐ Prescription of a anxiolytic drug
- ☐ Prescription of a psychotropic drug
- ☐ Referral to mental health or social services
  - ☐ Counsellor
  - ☐ Primary care mental health worker
  - ☐ Psychology
  - ☐ Social Services
  - ☐ Psychiatry
- ☐ No further action taken as patient known to suffer from pre-existing depression, anxiety or post traumatic stress disorder
- ☐ None of the above

Thank You!
