# Supplementary material for: Anxiety, depression and post-traumatic stress disorder management after critical illness: a UK multi-centre prospective cohort study
Source: Crit Care. 2020 Nov 2;24:633. doi: 10.1186/s13054-020-03354-y (PMC7607621; doi:10.1186/s13054-020-03354-y)
Supplement: Supplementary file 4 — Additional file 4 Management outcomes (Figure 2 as table). [file 13054_2020_3354_MOESM5_ESM.docx]

| **Timing from ICU discharge to first patient survey triggering GP notification** | **GP response rate** |
| --- | --- |
| 3 months | 65% |
| 12 months | 66% |

**Appendix E – GP Response rate following notification at 3 or 12 months following patient discharge from ICU**
